# Ultra-Low Dose Argatroban in Heparin-Induced Thrombocytopenia with Thrombocytopenia and Hypofibrinogenemia: A Case Report

**DOI:** 10.1055/a-2923-1469

**Published:** 2026-08-11

**Authors:** Chunqian Shi, Tong Cai, Mei Yue, Huijuan Ma, Qingfen Qin, Qian Song

**Affiliations:** 1 Department of Clinical Laboratory 594331 North China Medical and Health Group Xingtai General HospitalXingtaiHebei ProvinceChina

**Keywords:** heparin-induced thrombocytopenia, argatroban, thrombocytopenia, hypofibrinogenemia, coagulation–fibrinolysis markers

## Abstract

Heparin-induced thrombocytopenia (HIT) with thrombosis typically necessitates immediate therapeutic-dose anticoagulation, yet management becomes exceedingly complex when a profound bleeding risk coexists. We report a unique case of serologically confirmed HIT developing during low-molecular-weight heparin prophylaxis following major trauma in a 67-year-old man. On day 10 of heparin exposure, the patient presented with a platelet count declining from 169 × 10 ^9^ /L to 20 × 10 ^9^ /L (91% reduction), hypofibrinogenemia (1.12 g/L), extensive deep vein thrombosis, and pulmonary embolism. Owing to extreme bleeding risk, argatroban initiation was deferred for 48 hours postdiagnosis, then cautiously titrated from 0.08 to 0.67 μg/kg/min guided by dynamic coagulation–fibrinolysis markers (thrombin–antithrombin complex, plasmin–α2-antiplasmin complex, and D-dimer). These markers provided real-time assessment of treatment response, declining substantially prior to platelet count normalization. By day 12 postdiagnosis, platelet count recovered to 176 × 10 ^9^ /L, permitting successful definitive orthopedic surgery on day 13 without bleeding complications. This case illustrates that in HIT with life-threatening thrombosis and extreme bleeding risk, an individualized “start low, go slow” argatroban strategy with delayed initiation achieves effective anticoagulation while minimizing hemorrhagic risk.

## Introduction

Heparin-induced thrombocytopenia (HIT) is an immune-mediated adverse drug reaction triggered by platelet-activating immunoglobulin G antibodies targeting platelet factor 4 (PF4)–heparin complexes, resulting in thrombocytopenia and thrombosis. 1 Its prevalence ranges from 0.1% to 5% among heparin-exposed patients, varying with heparin type, patient population, and duration of exposure. 2 HIT typically manifests 5 to 10 days postheparin initiation, characterized by platelet count reduction exceeding 50% from baseline. 3 The hallmark complication is thrombosis, occurring in 30% to 75% of cases, encompassing deep vein thrombosis (DVT), pulmonary embolism (PE), and arterial thrombosis. 4

Argatroban, a direct thrombin inhibitor, is recommended as first-line treatment for HIT with thrombosis, with standard guidelines advising initial doses of 2.0 μg/kg/min. 5 In critically ill patients, 0.5–1.0 μg/kg/min is commonly recommended as the starting dose, with subsequent adjustment based on activated partial thromboplastin time (aPTT; target range, 1.5–3.0-fold baseline). 6 However, our patient’s initial dose of 0.08 μg/kg/min fell substantially below even these reduced recommendations, reflecting the extreme caution warranted when profound thrombocytopenia coexists with hypofibrinogenemia and extensive thrombosis—a scenario for which no specific guidance exists.

Herein, we describe a case of serologically confirmed HIT with extensive thrombosis but extreme bleeding risk in a trauma patient, demonstrating the feasibility and safety of individualized ultra-low dose argatroban titration guided by coagulation–fibrinolysis markers.

## Case

A 67-year-old previously healthy man (weight: 80 kg) was admitted on March 8, 2026, after sustaining a crush injury to his left ankle caused by a 500-kg iron block. He had no previous history of thrombosis, bleeding disorders, or exposure to heparin. Emergency management, conducted under combined spinal-epidural anesthesia, involved left ankle debridement, primary wound closure, vascular and nerve repair, tendon repair, and calcaneal traction.

**Fig. 1:**
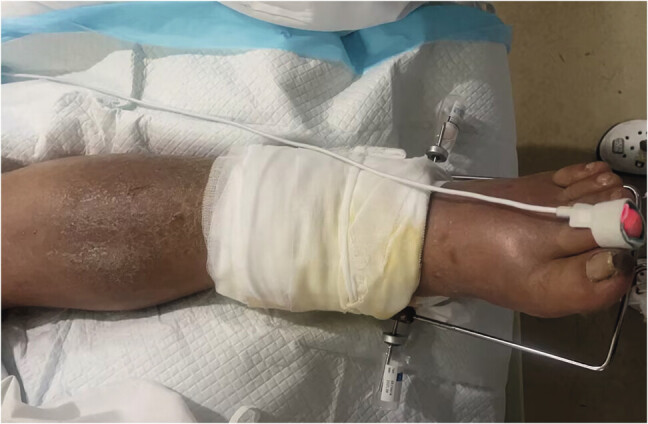
Clinical photograph of the left lower extremity on March 20, 2026, demonstrating marked swelling and skin changes consistent with extensive deep vein thrombosis in the setting of heparin-induced thrombocytopenia.

Radiography revealed comminuted fractures of the distal left tibia and fibula, characterized by overlapping displacement, multiple displaced bone fragments, and significant surrounding soft tissue edema. The clinical course is depicted in Fig. 1 . On March 18, 2026 (day 10 of heparin exposure), preoperative ultrasound identified extensive DVT in the left lower extremity, affecting the common femoral, deep femoral, superficial femoral, popliteal, great saphenous, and calf muscular veins. An inferior vena cava filter was placed on the same day.

Following the procedure, the patient experienced acute dyspnea with hemoptysis on March 19, 2026 (day 11), which was later confirmed to be due to PE by computed tomography angiography. On the same day, critical laboratory results showed a platelet count of 20 × 10 ^9^ /L (a decrease from 169 × 10 ^9^ /L on admission, representing a 91% reduction) and a fibrinogen level of 1.12 g/L.

The 4T score was calculated to be 6 points, indicating high probability: timing received 2 points (platelet count decline observed between days 5 and 10), thrombocytopenia received 2 points (platelet count nadir 20 × 10 ^9^ /L, with >50% decline from baseline), thrombosis received 2 points (new thrombosis confirmed), and other causes received 0 points (no alternative causes identified). 7 PF4-heparin IgG antibody testing yielded a positive result via enzyme-linked immunosorbent assay (optical density, >1.0). In conjunction with a 4T score of 6 points (high probability), a diagnosis of HIT was made with high clinical probability, and all heparin was discontinued immediately. 1

Initiation of argatroban was delayed until March 21, 2026 (day 2: postdiagnosis), because of severe thrombocytopenia (20 × 10 ^9^ /L) and hypofibrinogenemia (1.12 g/L), posing dual risks of thrombosis and hemorrhage necessitating meticulous risk assessment. The starting dose was 0.08 μg/kg/min, gradually increased to 0.13 μg/kg/min on March 22, 2026, 0.50 μg/kg/min on March 23, 2026, and 0.67 μg/kg/min on March 24, 2026, guided by dynamic changes in coagulation–fibrinolysis markers (thrombin–antithrombin complex [TAT], plasmin–α2-antiplasmin complex [PIC], and D-dimer), with aPTT being monitored but not utilized as the primary titration target. Notably, the maximum dose of 0.67 μg/kg/min remained significantly below the standard initial dose recommendation of 2.0 μg/kg/min, representing only a third of the typical starting dosage. On March 27, 2026 (post-HIT: day 8), argatroban administration was inadvertently halted due to a transient order entry mistake during inter-department transfer, leading to a decrease in aPTT to 40.21 s. Following error identification, the infusion was reinstated on March 28, 2026, at the prior dose of 0.67 μg/kg/min.

Due to the high risk of bleeding (platelet count of 20 × 10 ^9^ /L, fibrinogen level of 1.12 g/L) and the lack of clarity regarding the most effective anticoagulation intensity, we utilized coagulation–fibrinolysis markers to impartially evaluate the hypercoagulable condition. Sequential assessments were conducted for TAT, PIC, and D-dimer levels as detailed in Table 1 .

**Table 1:** Dynamic changes in coagulation–fibrinolysis markers, aPTT, and argatroban dose during treatment.

Date	Day ^a^	Argatroban (μg/kg/min)	TAT (ng/mL)	PIC (μg/mL)	D-dimer (μg/mL)	aPTT (s)
March 21, 2026	1	0.08	106.00	11.77	83.96	45.10
March 22, 2026	2	0.13	88.25	10.43	52.24	52.12
March 23, 2026	3	0.5	104.50	12.15	51.88	46.31
March 24, 2026	4	0.67	80.53	10.27	44.70	58.17
March 25, 2026	5	0.67	70.24	9.00	40.83	59.87
March 26, 2026	6	0.67	66.00	9.76	35.36	61.64
March 27, 2026 ^b^	7	0	67.77	12.44	46.4	40.21
March 28, 2026	8	0.67	30.08	4.68	44.31	60.91
March 29, 2026	9	0.67	20.81	1.91	36.85	62.39
March 30, 2026	10	0.67	14.49	1.37	16.40	61.37
March 31, 2026	11	0.67	13.08	1.26	11.60	58.31

Note: Reference ranges: TAT < 4 ng/mL; PIC < 0.8 μg/mL; D-dimer < 0.5 μg/mL.

^a^ Day after argatroban initiation (March 21, 2026 = day 1; first dose administered after baseline measurement).

^b^ Argatroban was inadvertently held on this day due to an order entry error.

On March 22, 2026 (day 2 after initiation of argatroban, 0.08 μg/kg/min), the morning TAT remained significantly elevated at 106.00 ng/mL (reference < 4), indicating substantial ongoing thrombin generation despite therapeutic anticoagulation. This observation, along with persistently high levels of PIC (11.77 μg/mL, reference < 0.8 μg/mL) and D-dimer (83.96 μg/mL), prompted cautious dose escalation to 0.13 μg/kg/min. By the evening of March 22, 2026, TAT decreased to 88.25 ng/mL but rebounded to 104.50 ng/mL on March 23, 2026, which supported further escalation to 0.50 μg/kg/min. On March 24, 2026, with the dose increased to 0.67 μg/kg/min, TAT initially declined to 80.53 ng/mL, justifying the maintenance of this relatively high dose despite the patient’s bleeding risk profile. Subsequently, the markers exhibited a progressive decline: TAT decreased to 70.24 ng/mL on March 25, 2026, and 66.00 ng/mL on March 26, 2026, followed by a slight rebound to 67.77 ng/mL. On March 27, 2026 (post-HIT: day 8), argatroban administration was unintentionally held due to an order entry error during inter-department transfer. After resuming the infusion, TAT fell sharply to 30.08 ng/mL by March 28, 2026, and continued to decline progressively: 20.81 ng/mL on March 29, 2026, 14.49 ng/mL on March 30, 2026, and approached near-normalization at 13.08 ng/mL by March 31, 2026, preceding platelet count recovery (176 × 10 ^9^ /L) and informing the decision to proceed with definitive surgery on April 1, 2026.

Throughout the patient’s admission, there was a gradual improvement in both clinical and laboratory parameters. The platelet count progressively recovered, reaching 176 × 10 ^9^ /L by March 31, 2026 (day 12: postdiagnosis). Coagulation–fibrinolysis markers significantly decreased from their peak values but remained mildly elevated above normal ranges on day 12, with TAT at 13.08 ng/mL (reference < 4), PIC at 1.26 μg/mL (reference < 0.8), and D-dimer at 11.60 μg/mL. The aPTT consistently remained below 1.5-fold the baseline value, measuring 45.10 s on March 21, 2026, after heparin discontinuation and prior to argatroban initiation. It peaked at 60.91 s on March 28, 2026, and reached 62.39 s (1.38-fold) on March 29, 2026. Given the significant risk of bleeding, the conventional aPTT target of 1.5 to 3.0-fold the baseline was intentionally not pursued. Instead, dose titration was primarily guided by the dynamics of TAT, PIC, and D-dimer to strike a balance between thrombotic control and hemorrhagic safety.

The patient underwent definitive orthopedic surgery on April 1, 2026, which involved open reduction and internal fixation of tibial and fibular fractures using external fixation and vacuum sealing drainage. The procedure was conducted under combined spinal-epidural anesthesia, following the restoration of the platelet count to 176 × 10 ^9^ /L and the normalization of fibrinogen levels, which occurred on day 13 after the diagnosis of HIT. Postoperatively, argatroban was continued until transition to oral rivaroxaban once platelet count stabilized above 150 × 10 ^9^ /L and gastrointestinal function permitted.

The patient was discharged with no further anticoagulation-related complications. Laboratory parameters remained stable at short-term follow-up.

## Discussion

HIT is associated with diverse clinical presentations, with thrombocytopenia as its hallmark. Central to its pathophysiology is formation of PF4–heparin immune complexes that activate platelets and generate procoagulant microparticles, culminating in a paradoxical hypercoagulable state. 1 In our patient, this immune-mediated prothrombotic drive coexisted with profound thrombocytopenia and hypofibrinogenemia, creating competing thrombotic and hemorrhagic risks that precluded standard-dose anticoagulation.

Current guidelines suggest reduced argatroban doses for specific populations; however, our patient’s initial dose of 0.08 μg/kg/min reflected a scenario—profound thrombocytopenia with hypofibrinogenemia and extensive thrombosis—for which no specific guidance exists. In this patient, competing thrombotic and hemorrhagic risks coexisted: thrombosis from active HIT with extensive DVT/PE, 4 and bleeding risk from profound thrombocytopenia and hypofibrinogenemia, potentially exacerbated by trauma-induced coagulopathy. This combination precluded standard-dose anticoagulation.

Prior evidence has documented successful argatroban use at markedly reduced doses in critically ill patients. 8 Our case extends these observations in three key aspects: first, continuous infusion at 0.08 μg/kg/min—well below the lowest guideline-recommended reduced dose—proved feasible and effective; second, dose titration to 0.67 μg/kg/min over 4 days was guided primarily by coagulation–fibrinolysis marker dynamics rather than aPTT targets, reflecting a strategy of balancing thrombotic control with hemorrhagic safety in extreme bleeding risk; third, serial measurements of TAT, PIC, and D-dimer provided real-time assessment of coagulation activation, fibrinolytic activation, and fibrin degradation, respectively. This enabled dynamic dose adjustment throughout the treatment course.

TAT and PIC are emerging biomarkers reflecting thrombin generation and fibrinolytic activation, respectively, with demonstrated diagnostic and therapeutic monitoring value in DVT/VTE settings. 9 10 Their application in HIT has not been specifically validated in prospective studies; however, given that HIT pathophysiology centers on uncontrolled thrombin generation, we hypothesized that these markers might provide mechanistically plausible monitoring in this context, pending formal evaluation. In our case, markedly elevated baseline TAT (106.00 ng/mL), PIC (11.77 μg/mL), and D-dimer (83.96 μg/mL) confirmed intense hypercoagulability and active fibrinolysis despite profound thrombocytopenia, while their progressive decline toward near-normalization by day 12 (TAT: 13.08 ng/mL; PIC: 1.26 μg/mL; D-dimer: 11.60 μg/mL) provided objective evidence of effective anticoagulation. These dynamic changes guided dose escalation during the acute phase and supported the decision to proceed with surgery once markers approached normalization.

This case is distinctive in that it presents a rare clinical scenario of competing thrombotic and hemorrhagic risks in HIT. Extensive thrombosis, profound thrombocytopenia, and hypofibrinogenemia coexisted in a single patient, managed successfully with individualized ultra-low dose argatroban titration guided by dynamic biomarker monitoring. The patient recovered favorably, and at 1-month follow-up remained in good health without recurrent thrombotic events.

## Limitations

This single-case observation precludes generalizability, and the optimal TAT/PIC target ranges for argatroban titration in HIT remain undefined.

## Conclusion

This case demonstrates that serologically confirmed HIT with extensive thrombosis can be successfully managed with individualized ultra-low dose argatroban titration guided by dynamic coagulation–fibrinolysis markers (TAT, PIC, and D-dimer) in the setting of profound thrombocytopenia and hypofibrinogenemia. The “start low, go slow” strategy achieved effective anticoagulation without bleeding complications, highlighting that dynamic biomarker monitoring may enable individualized dosing that balances thrombotic control with hemorrhagic safety when standard monitoring targets are intentionally deferred.

## Data Availability

The data supporting this case report are available from the corresponding author upon reasonable request, subject to patient confidentiality protections.

## References

[1] Arepally G M Heparin-induced thrombocytopenia Blood 2017 129 21 2864 2872 28416511 10.1182/blood-2016-11-709873 PMC5445568

[2] Hogan M Berger J S Heparin-induced thrombocytopenia (HIT): review of incidence, diagnosis, and management Vasc Med 2020 25 02 160 173 32195628 10.1177/1358863X19898253

[3] Greinacher A Farner B Kroll H Kohlmann T Warkentin T E Eichler P Clinical features of heparin-induced thrombocytopenia including risk factors for thrombosis. A retrospective analysis of 408 patients Thromb Haemost 2005 94 01 132 135 16113796 10.1160/TH04-12-0825

[4] Warkentin T E Heparin-induced thrombocytopenia: pathogenesis and management Br J Haematol 2003 121 04 535 555 12752095 10.1046/j.1365-2141.2003.04334.x

[5] Cuker A Arepally G M Chong B H American Society of Hematology 2018 guidelines for management of venous thromboembolism: heparin-induced thrombocytopenia Blood Adv 2018 2 22 3360 3392 30482768 10.1182/bloodadvances.2018024489 PMC6258919

[6] Selleng K Warkentin T E Greinacher A Heparin-induced thrombocytopenia in intensive care patients Crit Care Med 2007 35 04 1165 1176 17334253 10.1097/01.CCM.0000259538.02375.A5

[7] Cuker A Gimotty P A Crowther M A Warkentin T E Predictive value of the 4Ts scoring system for heparin-induced thrombocytopenia: a systematic review and meta-analysis Blood 2012 120 20 4160 4167 22990018 10.1182/blood-2012-07-443051 PMC3501714

[8] Saugel B Phillip V Moessmer G Schmid R M Huber W Argatroban therapy for heparin-induced thrombocytopenia in ICU patients with multiple organ dysfunction syndrome: a retrospective study Crit Care 2010 14 03 R90 20487559 10.1186/cc9024 PMC2911727

[9] Chen Q Shou W Wu W Wang G Cui W Performance evaluation of thrombomodulin, thrombin-antithrombin complex, plasmin-α2-antiplasmin complex, and t-PA: PAI-1 complex J Clin Lab Anal 2019 33 06 e22913 31090232 10.1002/jcla.22913 PMC6642299

[10] Lin Z Sun H Li D Thrombin antithrombin complex concentration as an early predictor of deep vein thrombosis after total hip arthroplasty and total knee arthroplasty BMC Musculoskelet Disord 2022 23 01 574 35701797 10.1186/s12891-022-05532-1 PMC9195246

